# Fever and infections in surgical intensive care: an American Association for the Surgery of Trauma Critical Care Committee clinical consensus document

**DOI:** 10.1136/tsaco-2023-001303

**Published:** 2024-06-03

**Authors:** Eden Nohra, Rachel D Appelbaum, Michael Steven Farrell, Thomas Carver, Hee Soo Jung, Jordan Michael Kirsch, Lisa M Kodadek, Samuel Mandell, Aussama Khalaf Nassar, Abhijit Pathak, Jasmeet Paul, Bryce Robinson, Joseph Cuschieri, Deborah M Stein

**Affiliations:** 1 Department of Surgery, University of Colorado Anschutz Medical Campus, Aurora, Colorado, USA; 2 Department of Surgery, Vanderbilt University Medical Center, Nashville, Tennessee, USA; 3 Department of Surgery, Lehigh Valley Health Network, Allentown, Pennsylvania, USA; 4 Department of Surgery, Medical College of Wisconsin, Milwaukee, Wisconsin, USA; 5 Department of Surgery, University of Wisconsin Madison School of Medicine and Public Health, Madison, Wisconsin, USA; 6 Department of Surgery, Westchester Medical Center/ New York Medical College, Valhalla, NY, USA; 7 Department of Surgery, Yale University School of Medicine, New Haven, Connecticut, USA; 8 Department of Surgery, The University of Texas Southwestern Medical Center, Dallas, Texas, USA; 9 Department of Surgery, Section of Acute Care Surgery, Stanford University, Stanford, California, USA; 10 Department of Surgery, Temple University School of Medicine, Philadelphia, Pennsylvania, USA; 11 Department of Surgery, University of New Mexico Health Sciences Center, Albuquerque, New Mexico, USA; 12 Department of Surgery, Harborview Medical Center, Seattle, Washington, USA; 13 Department of Surgery, Zuckerberg San Francisco General Hospital and Trauma Center, San Francisco, California, USA; 14 Department of Surgery, University of Maryland Baltimore, Baltimore, Maryland, USA

**Keywords:** critical care, infections

## Abstract

The evaluation and workup of fever and the use of antibiotics to treat infections is part of daily practice in the surgical intensive care unit (ICU). Fever can be infectious or non-infectious; it is important to distinguish between the two entities wherever possible. The evidence is growing for shortening the duration of antibiotic treatment of common infections. The purpose of this clinical consensus document, created by the American Association for the Surgery of Trauma Critical Care Committee, is to synthesize the available evidence, and to provide practical recommendations. We discuss the evaluation of fever, the indications to obtain cultures including urine, blood, and respiratory specimens for diagnosis of infections, the use of procalcitonin, and the decision to initiate empiric antibiotics. We then describe the treatment of common infections, specifically ventilator-associated pneumonia, catheter-associated urinary infection, catheter-related bloodstream infection, bacteremia, surgical site infection, intra-abdominal infection, ventriculitis, and necrotizing soft tissue infection.

## Introduction

As clinicians and intensivists, we strive to diagnose and treat infection. Fever occurs commonly in the surgical intensive care unit (ICU), but the etiology is infectious only half the time. In the management of infections, the right treatment and duration is an essential component of critical care management. In this clinical consensus document, the AAST Critical Care Committee aims to provide practical guidance to the surgical intensivist on the best practices in the evaluation of fever and the treatment of infections in the adult, age ≥16 years of age, critically ill and injured patient.

## Methods

The AAST Critical Care Committee chose antibiotic management in the ICU as a clinically relevant topic for review. This document is one of a three-part series on this topic (Appelbaum, TSACO (in submission), Farrell, TSACO (in submission)). The subtopics reviewed are not comprehensive for the topic of antibiotic management in the ICU but were specifically selected to be practical and useful for the surgical intensivist. A working group was formed from the committee at large to complete this work. The members of the working group were each assigned a subtopic to review using research to date. The members were asked to base their recommendations on research within the last 10 years. If research is unique, important, and has not been replicated, then it may be used even if it is older than 10 years. The research on which the recommendations are based was compiled at the discretion of the working group. Iterative selection of studies was not performed as in a systematic review, and the methodology of the literature search was at the discretion of the authors. The recommendations were then reviewed by the AAST Critical Care Committee at large. Consensus was either achieved by conference or reported as ‘no consensus’. The recommendations apply to adult trauma patients, aged ≥16 years of age. Clinicians must take into account other considerations such as weight and pregnancy for adjustments in dosing and specific antibiotic selection.

## Disclaimer from the AAST Critical Care Committee

The work represents expert opinion and the recommendations of the entire committee. These recommendations do not intend to substitute for the provider’s clinical experience. The intent of the AAST Critical Care Committee clinical consensus documents is to provide healthcare professionals with evidence-based recommendations regarding care of the critically ill patient. The clinical consensus documents do not include all potential options for prevention, diagnosis, and treatment, and they are not intended as a substitute for the provider’s clinical judgment and experience. The responsible provider must make all treatment decisions based on their independent judgment and the patient’s individual clinical presentation. The AAST and any entities endorsing the clinical consensus document shall not be liable for any direct, indirect, special, incidental, or consequential damages related to the use of the information contained here. The AAST may modify the clinical consensus documents at any time without notice.

## ICU fever

### Question

How is fever in the ICU assessed and defined?

### Recommendation

A temperature >38.3°C in critically ill patients is defined as a fever,^1^ and >39.5°C as a high fever, except in neutropenia. It is important to consider that, in the elderly, the fever response may be blunted and thus, an infected elderly person may not manifest a fever. Additionally, certain ICU conditions and treatments can easily mask fever, as discussed below.

### Discussion

Therapies such as continuous renal replacement therapy (CRRT), peritoneal lavage, or extracorporeal membrane oxygenation (ECMO) may alter core temperature. Environmental considerations such as room temperature, mattress type, lights, and external warming devices may also impact core body temperature. Clinicians should consider patient-specific factors when evaluating temperature data in a critically ill patient. Furthermore, not all patients with infection will generate a fever: the elderly, those with open abdominal wounds or large total body surface area burns, patients treated with antipyretics, or those on ECMO or CRRT may be euthermic or hypothermic.^2^ Fever in patients with neutropenia (absolute neutrophil count <500 cells/mm) is defined as temperature ≥38.0°C sustained over 1 hour or recurrent over 12 hours.^3^

In the elderly, a lower cut-off for fever is considered specifically for older adult residents of long-term care facilities.^4^ The definition has not been extended to critically ill older adults as evidenced by the joint guidelines of the Society of Critical Care Medicine (SCCM) and Infectious Disease Society of America (IDSA) on the workup of fever written in 2023.^1^ However, and importantly so, other signs of infection besides fever should be closely evaluated as it is common that an infected elderly person does not manifest a fever due to blunted physiological responses.^5 6^ In older adult patients, change in behavior, a rise in baseline temperature by 1 degree, lack of cooperativeness with care, laboratory values that indicate organ dysfunction, altered mentation, and change from baseline including fatigue, loss of appetite, delirium, and falls should all be considered possible signs of an infection.^6 7^

Devices used should be assessed, maintained, and calibrated regularly according to manufacturer’s guidelines. Temperature is most accurate when measured with esophageal probes and bladder catheter thermistors (as opposed to axillary or tympanic), however central measurement is not always necessary.^8^

### Question

What is the recommended approach to a patient with fever in the ICU?

### Recommendation

**Figure 1:**
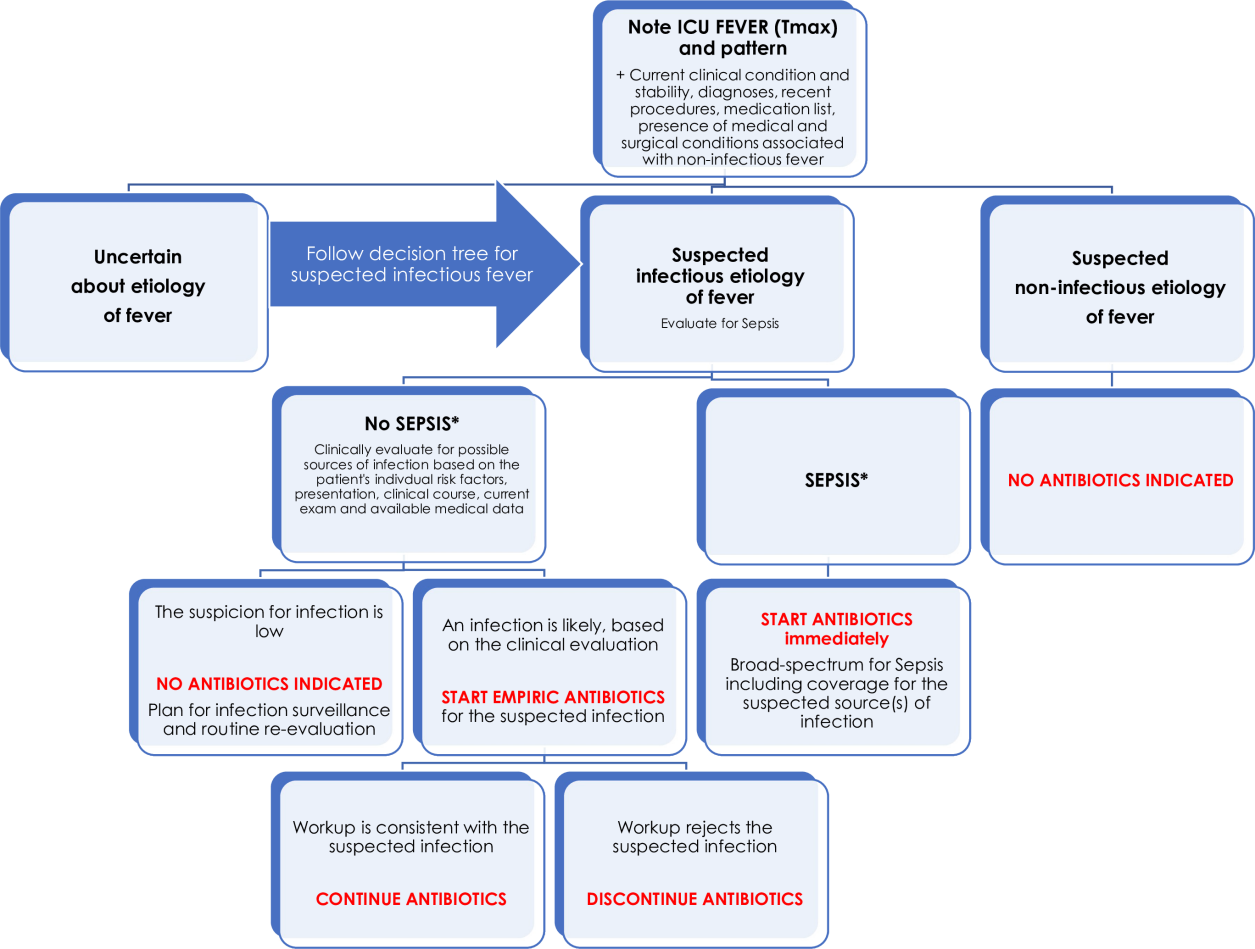
Flow chart for intensive care unit (ICU) fever and antibiotic management. *Defined as sepsis 3—life-threatening organ dysfunction caused by a dysregulated host response to infection where organ dysfunction is represented by an increase in Sequential Organ Failure Assessment (SOFA) score by 2 points or the patient has septic shock.^17^

A comprehensive differential diagnosis for fever must be considered, weighing all causes of fever including infectious and non-infectious etiologies. A targeted workup guided by clinical and physical evaluation should be ordered, and close re-evaluation should be performed for escalation, de-escalation, or discontinuation of the treatment regimen. Please see approach to fever in the ICU in figure 1.

### Discussion

While infections are a common occurrence in the ICU patient, any process that causes a release of inflammatory cytokines can lead to fever.^9^ This is important in the surgical ICU because tissue injury is a well-known cause of fever and up to 39% of postoperative patients will have at least one febrile episode.^10 11^ The pattern of fever may be helpful in distinguishing certain pathologies. For instance, non-infectious causes are associated with temperatures <38.9°C while extreme temperatures (>41°C) are almost never infectious.^9^ Extreme temperatures raise concern for neuroleptic malignant syndrome, drug fever, or malignant hyperthermia.^12^ Alternatively, temperatures >39.3°C, especially if they persist for several days, are more likely infectious.^13^

Non-infectious causes of fever are often overlooked due to the overwhelming concern for a bacterial source.^13 14^ Some of these non-infectious causes are listed in table 1. Therapies used may cause fever, such as drug fevers especially in the presence of a rash. Some drugs commonly implicated in fever in the ICU are listed in table 2. In addition, temperature variation occurs frequently in critical illness due to altered circadian rhythms and autonomic dysfunction.^10^ While fever itself is a poor predictor of positive cultures,^11^ it is highly associated with obtaining cultures (OR 3.8 in one study) which underscores the fact that fever does not equate with infection.^15^

**Table 1:** Non-infectious causes of fever in the intensive care unit

System	Etiology
Cardiovascular	Deep venous thrombosis
	Pericarditis
	Myocardial infarction
	Thrombophlebitis
Neurological	Stroke
	Traumatic brain injury
	Seizure
	Intracranial hemorrhage
Endocrine	Adrenal insufficiency
	Hyperthyroidism
Gastrointestinal	Acalculous cholecystitis
	Ischemic bowel
	Hepatitis
	Pancreatitis
Respiratory	Acute respiratory distress syndrome
	Pneumonitis
	Pulmonary embolism
Other	Autoimmune disorders
	Blood product transfusion
	Drug/Alcohol withdrawal
	Drug fever
	Fat embolism
	Hematoma
	Malignancy
	Postoperative fever

**Table 2:** Common medications associated with drug fever

Antibiotics	Βeta-lactams
	Sulfonamides
	Nitrofurantoin
Antiarrhythmics	Procainamide
	Quinidine
Anti-epileptics	Phenytoin
	Carbamazepine
Sedatives	Dexmedetomidine
	Barbiturates
Sulfa-containing	Loop diuretics
	Stool softeners

In surgical and neurological ICUs, respiratory infections account for the majority of infectious fevers.^14 16^ Postoperative patients are at an obvious risk for certain infections, including skin and soft tissue infections,^9^ *Clostridium difficile* colitis, central line-associated bloodstream infections; and rarely, catheter-associated urinary tract infections (CAUTIs).^10 17^ Certain patient populations have additional infection risk exposures such as ventriculitis in neurosurgical patients or those with open brain injury.

The widespread application of ‘pan-culture’ for fever has come into question as it is associated with increased antibiotic use without added clinical benefit and with significant harms,^18^ including increased antibiotic use, costs, patient discomfort, and iatrogenic infections.^13 19^ On the other hand, since the diagnostic accuracy of clinical exam alone is lacking (60% sensitive and 64% specific), we recommended that the clinical evaluation be supplemented with additional tests selectively guided by the clinical suspicion, patient symptomatology, and/or risk for certain infections.^10 18^ We are reassured that delaying antibiotics for a period of hours until relevant workup has returned or the workup and evaluation for fever has further developed does not worsen outcome.^20^ We therefore encourage a comprehensive evaluation of the patient, including risk factors, for all potential sources of fever (including non-infectious sources) and weighing this carefully with the developing clinical condition prior to any decision for antibiotic initiation and prior to subjecting the patient with fever to a broad panel of cultures.^11 17^ The caveat is that the clinician should have a heightened awareness for the true definition of sepsis (new organ dysfunction resulting from an infection), which would necessitate immediate antibiotic treatment, source evaluation, and control.^21^

## Cultures in the evaluation of a fever

### Urine

#### Question

In the workup of fever, when should urinalysis and culture be obtained?

#### Recommendation

The absence of urinary symptoms in the correct clinical setting should obviate the need for urinalysis and culture, regardless of the presence or absence of a catheter. Fever alone should never trigger urine studies. In a patient with sepsis (per sepsis-3 guideline definition)^22^ or septic shock, a decision can be made to obtain urinalysis and culture provided the source of sepsis is determined to be unclear after careful evaluation by the ICU.

#### Discussion

Decades of surgical dogma has led to the pervasive belief that the urine should be evaluated in a patient with fever, particularly if a catheter is present. This practice is misguided given that pyuria and bacteriuria are frequently present in patients with urinary catheters in the absence of clinical infection.^23^ No cut-offs for the degree of leukocytosis or fever have even been found to correlate with UTI.^24^ Urinary workup in the absence of appropriate concern for UTI is both costly and leads to unnecessary antibiotics.^25 26^ Most of the time, an alternative cause of the fever is identified.^27^ When urinalysis and cultures are done, one will find bacterial growth about half the time which may be indistinguishable from colonization. We do not encourage routine urinalysis and culture for screening because these may incur unintended harms including unnecessary treatment.^28^ Colonization of the urine rarely develops into urosepsis, but it is possible after a urological procedure or other urological abnormality so testing and diagnosis of UTI may be differently nuanced in this setting.^12 13^ Furthermore, we make no comment on straight catheterization versus indwelling catheterization because there is no convincing evidence of decreased risk of infection with intermittent straight catheterization even in patients with spinal injury.^29^

Clinical risk factors that should raise concern for UTI include previous episodes, urological procedure, abnormal urological anatomy, neutropenia, kidney transplant, and urinary obstruction. The use of multidisciplinary input and algorithms to determine likelihood of UTI are useful alongside the evaluation of fever prior to initiating urinalysis and cultures.^30^ Especially in patients who cannot display symptoms, urinalysis and culture can be part of an evaluation for sepsis when the sepsis is determined, after evaluation by the ICU, to be without a clear source. If the patient can be alert enough, urinary symptoms must be assessed including flank pain and pelvic discomfort. Discoloration, odor, and consistency of urine or any kind of change in its appearance are not considered symptoms of UTI.^28^ Finally, in the ICU, the chance that colonization and other abnormal urinalysis results will be misclassified and diagnosed as CAUTI is unacceptably high, which reinforces the need to abandon the old surgical dogma of obtaining a urinalysis for every fever.^31 32^

### Blood

#### Question

In the workup of fever, when should blood cultures be obtained?

#### Recommendation

Initial blood cultures are needed for conditions associated with bacteremia including necrotizing skin and soft tissue infections, meningitis, systemic infection associated with asplenia and severe intra-abdominal infections (IAIs). Fever alone should not trigger blood cultures. A localized infection should not trigger blood cultures. Sepsis (per sepsis-3 guideline definition) and/or septic shock can trigger blood cultures. Repeat blood cultures are not routine but may be clinically warranted in certain situations. Blood cultures can be considered if the risk to the patient is high if a bacteremia is missed. If a patient has a central line and blood cultures are warranted based on the clinical evaluation, then the diagnosis of catheter-related bloodstream infection (CRBSI) should be made where appropriate.

#### Discussion

Neither normothermia nor the presence of fever correlate with bacteremia.^11^ Similarly, the combination of leukocytosis and fever has no correlation to bacteremia.^33^ Finally, contrary to popular belief, arterial lines appear to have the same risk as central lines for bloodstream infections.^11 34^ Testing stewardship is important, in part because the rate of false positive cultures from contamination can be as high as 50%.^35 36^ The likelihood of bacteremia based on the clinical judgment of pretest probability should guide the decision to draw blood cultures.^37^ Ordering blood cultures should be predicated on the nature and severity of the suspected infection.

The importance of obtaining blood cultures during a febrile episode is overemphasized, even when a central line is present.^5^ If a patient has sepsis (per sepsis-3 guidelines) or septic shock or if the source of their suspected infection is associated with a high rate of bacteremia, then they may need blood cultures. If new blood cultures are being considered, a new physical examination and evaluation of the patient’s likely diagnosis and condition need to be made prior to this decision. It is important to recall that if blood cultures return negative in a patient with sepsis, this should not give the clinician reassurance about their condition.^38^

There is no role for routine surveillance blood cultures. Routine repeat blood cultures to assess clearance of bacteremia are usually not needed except if the patient does not clinically improve or if they are at risk for metastatic infections (eg, *Staphylococcus aureus*).^39–41^ Blood cultures can be considered if the risk to the patient is high if a bacteremia is missed (eg, in a patient with a pacemaker and cellulitis).^39^

Regarding culturing methods, separate fungal cultures are not needed because most *Candida* species grow better in normal culture media.^42^ Two sets of blood cultures yield the most reasonable data with sensitivity and specificity for true bacteremia. When faced with a positive blood culture, it is important to use multidisciplinary support to differentiate contaminant from true bacteremia. If a central line is present for >48 hours and the infection is not attributable to a different source, then the diagnosis of CRBSI must be entertained in a multidisciplinary fashion.^43^

### Respiratory

#### Question

In the workup of fever, when should respiratory cultures be obtained?

#### Recommendation

The lack of evidence of a new clinical syndrome of pneumonia should obviate the need for a respiratory specimen. Fever alone should not trigger respiratory cultures.

#### Discussion

Pulmonary infections are one of the most common causes of fever in critically ill patients, affecting an estimated 25%–33% of ICU patients, this is more common in trauma and the risk is increased in certain injury patterns and with increased injury severity.^9 44^ Neither fever nor leukocytosis, nor the combination are associated with positive respiratory cultures, but they are frequently obtained even in the absence of X-ray findings or clinical evidence of pneumonia.^45 46^ Respiratory cultures may help support the diagnosis, but the presence of bacteria on culture is not diagnostic of a pneumonia because a majority of intubated patients will have colonization of the endotracheal tube—this is especially true if tracheal aspirates are used, although the use of bronchioalveolar lavage (BAL) does not eliminate false positives or false negatives.^18 47^ Unfortunately, a positive culture is often routinely managed with antibiotics regardless of the diagnostic impression.^25 48^

The clinical determination or strong suspicion of the syndrome of pneumonia should guide whether or not cultures are initiated. Information for this determination includes imaging findings (chest X-ray, ultrasound or CT), new or acutely worsened oxygenation deficit, the onset of purulent secretions, with concomitant new fever or white count that is not otherwise explained. We find that the clustering of factors in the correct clinical setting is more useful than a single score or numerical cut-off.

There is no strong data to support BAL, mini-BAL, or protected specimen brushing over non-invasive methods of tracheal aspiration or for semi-quantitative over qualitative cultures.^49^ An argument can be made for or against either. The joint guideline from the American Thoracic Society (ATS) and the Infectious Diseases Society of America (IDSA) weakly recommends non-invasive sampling based on low-quality evidence, while in trauma patients the utility of mini-BAL has been demonstrated specifically in its ability to parse the diagnosis of pneumonia from acute respiratory distress syndrome (ARDS).^44^ We recommend institutional multidisciplinary review of accepted practices and verification of correct interpretation based on techniques used.

#### Question

When is it appropriate to hold antibiotics in cases of fever in the ICU?

#### Recommendation

Due to significant harm associated with inappropriate antibiotic therapy, it is important to evaluate the likelihood of infection when deciding for or against empiric antibiotic initiation. Once started, de-escalation or stoppage should occur in a timely manner with decision-support by multidisciplinary evaluation and local protocols. Procalcitonin can be used in the context of a multidisciplinary institutional protocol, however, the utility is limited in critically injured patients and certain surgical populations.

#### Discussion

The benefits and detriments of antibiotic use, especially in patients without an infection, are not clearly understood; however, antibiotic exposure has been associated with an increased risk of subsequent infections, increased length of stay, and increased mortality.^25 50^ It is therefore imperative to closely examine the likelihood of infection in a patient prior to antibiotic initiation taking into context the entire clinical presentation and clinical trajectory.

Prompt re-evaluation and discontinuation of ineffective therapies is important.^25^ Intentionally withholding antibiotics may have a benefit when appropriate care is otherwise provided.^20^ De-escalation, or stopping antibiotics altogether, should be done once cultures are finalized because this practice both decreases bacterial resistance and lowers 90-day mortality.^10^

Procalcitonin has been shown to significantly reduce antibiotic use for lower respiratory infections without adversely impacting outcome.^51 52^ It has a high negative predictive value of 91%^40^ and follow-up levels have been shown useful for antibiotic discontinuation,^18^ however, caution is advised in circumstances that raise procalcitonin at baseline such as trauma including surgical trauma, and inflammatory conditions, like pancreatitis. There is no standard recommended use of procalcitonin in the critically ill trauma population.^53^

## ICU infections

### Ventilator-associated pneumonia

#### Question

What is the appropriate treatment approach for ventilator-associated pneumonia (VAP)?

#### Recommendation

Initiation of broad-spectrum antibiotics for VAP requires consideration of patient-specific culture data, recent antibiotic exposure, the local antibiogram, and timing of when infection developed. Common regimens for hospital-acquired infections are vancomycin plus either cefepime or piperacillin-tazobactam or in cases of severe penicillin allergy, aztreonam, although no specific regimen is generally superior. Empiric anaerobic coverage is not routinely recommended. We recommend de-escalation of antibiotic treatment when culture data are available. Seven days of treatment is sufficient for most patients. Methicillin-resistant *S. aureus* (MRSA) nasal swab testing should be used to determine the need for empiric coverage.

#### Discussion

Evidence suggests mortality is lower when the initial antibiotic therapy is effective, even when switched to adequate therapy after culture data become available.^49^ Therefore, it is important to initiate appropriate antibiotics when there is strong clinical suspicion of VAP. There is no evidence of superiority of one specific empiric regimen over another and appropriateness is targeted in context of the hospital antibiogram and specific patient risk factors.

Trauma is a risk factor for staphylococcal infections, specifically traumatic head injury and road traffic injuries.^54 55^ MRSA nasal swab testing is useful because of its high negative predictive value for MRSA carrier status and if negative can obviate the need for MRSA coverage, as evidenced by recent data in the trauma population.^56 57^ Anaerobic coverage is not routinely recommended due to lack of evidence of benefit and some evidence of harm.^58 59^ Empiric coverage is specifically tailored to *S. aureus*, *Pseudomonas*, and Gram-negative bacilli.

High-level evidence shows no benefit with treating longer than 7 days in most patients with exceptions limited to severe lung disease, severe immunosuppression, concomitant ARDS, and multidrug resistance.^60 61^ To de-escalate an antibiotic regimen, it is important that culture data be obtained at the time of diagnosis. Duration of therapy of 7 days and antibiotic de-escalation recommendations are consistent with the ATS/IDSA guidelines of 2016.^49^

### Catheter-associated urinary tract infection

#### Question

What is the appropriate treatment of CAUTI in the critically ill patient?

#### Recommendation

Treatment for CAUTI should be targeted to the likely causative organisms, local antibiograms, and patient risk factors. In complicated UTI, 7 days of piperacillin-tazobactam, or meropenem if the risk of extended-spectrum beta-lactamase (ESBL) producers is high. Seldom are longer courses needed unless there is no symptomatic improvement within the first few days (then 10–14 days are required). The catheter should be removed or exchanged wherever possible.

#### Discussion

Note that the diagnosis of CAUTI should not be made on urinalysis alone and a positive urinalysis without symptoms or sepsis (per sepsis-3 guidelines) should not trigger treatment. Upper urinary tract symptoms include flank pain, costophrenic angle tenderness, shaking fever or chills, severe systemic symptoms. Choice of antibiotic will depend on clinical severity, previous antibiotic use, risk of resistant organisms, and clinical risk of deterioration, and local antibiograms. De-escalation of antibiotic treatment should also occur based on culture data. The catheter should be removed or exchanged wherever possible at the time infection is first suspected.^62^ Note that protocolized urine sampling, such as requiring a culture via new urine catheter or straight catheterization, has reduced the rate of CAUTI infection diagnosis by reducing the risk of contamination by colonization,^63^ however, it is unlikely that this practice completely eliminates colonization from the urinary specimen. There has been no update to the IDSA guidelines or significant new data since 2009.^64^

### Catheter-related bloodstream infection

#### Question

What is the most effective approach and antibiotic therapy for the management of CRBSI?

#### Recommendation

Effective management of CRBSI involves timely diagnosis, prompt removal of vascular access if at all possible (source control), and appropriate antibiotic therapy for 7–14 days depending on the causative microorganism, as shown in table 3. Vancomycin plus a beta-lactam (such as piperacillin-tazobactam or a ceftazidime) is usually a good empiric regimen if the risk of ESBL is not high. Reference to the local antibiogram and hospital recommendations is recommended for the selection of empiric therapy.

**Table 3:** Summary of antibiotic durations for common ICU infections

Infection	Antibiotic recommendations	Comments
CAUTI*	3–7 days of antibiotic therapy is sufficient for most patients 10–14 days if symptoms do not improve early in the course	3 days is considered in age <65 years, and mild infection with no upper tract symptoms, and catheter has been removed. If treating with levofloxacin, consider 5 days only if patient is not severely ill.
VAP	7 days of antibiotic therapy	In ARDS or structural lung disease with virulent or resistant infections, 10–14 days may be considered. In severe immunocompromise such as organ transplant, 10–14 days may be considered.
CRBSI	7 days of antibiotic therapy for GNBs or coagulase-negative staphylococci 14 days if *Staphylococcus aureus* (and uncomplicated infection) or *Candida*	If endocarditis or other complicated form of CRBSI, 4–6 weeks may be required.
Bacteremia	7 days of antibiotic therapy for GNBs or coagulase-negative staphylococci 14 days if *S. aureus* (and uncomplicated infection) or *Candida*	It must be kept in mind to rule out an underlying source of the bacteremia.
Intra-abdominal infection†	3–5 days when source control is present 5–7 days and then re-evaluate when source control is not present	
Surgical site infection	1–2 days if significant cellulitis or systemic symptoms	The tenant of treatment is drainage of the infected material.
Ventriculitis	10–14 days of antibiotic therapy 21 days if *S. aureus*	
Necrotizing skin and soft tissue infection	2–4 days after final surgical debridement provided certain conditions are met (see section in text)	

*The recommendations and comments are in agreement with the IDSA guidelines.^64^

†The recommendations are in agreement with the SIS guidelines.^84^

CAUTI, catheter-associated urinary tract infection; CRBSI, catheter-related bloodstream infection; GNBs, Gram-negative bacilli; ICU, intensive care unit; SIS, Surgical Infection Society; VAP, ventilator-associated pneumonia.

#### Discussion

The diagnosis of CRBSI should be distinguished from secondary bacteremia due to other sources.^65^ Surveillance cultures for patients with central lines are not recommended when CRBSI is not suspected such as in an asymptomatic patient and should not be done when other cultures are more appropriate to evaluate for the clinically suspected infection (eg, respiratory cultures for a suspected pneumonia).^66^

The choice of antibiotic therapy should be based on local susceptibility patterns and the severity of illness and should be de-escalated when culture data become available. The recommended duration of antibiotic therapy is 7 days for coagulase-negative staphylococci, 7 days for Gram-negative bacilli,^67–69^ 14 days for *S. aureus* (unless a complicated infection is present)^41^; and 14 days for *Candida* (in the absence of retinitis or risk factors for it, as described in the ‘Bacteremia’ section). Examples of when a *S. aureus* infection is considered complicated include endocarditis, osteomyelitis, foreign body or implant, metastatic infection, low minimum inhibitory concentration (MIC), immunocompromise, and recurrent infection. An infectious disease consultation should be sought in *S. aureus* CRBSI.

The management described here applies to non-tunneled lines. When a CRBSI is suspected, the central line should be removed.^70^ Catheter salvage options when the line cannot reasonably be removed are beyond the scope of this text.

### Bacteremia

#### Question

What is the management of bacteremia in the critically ill patient?

#### Recommendation

Management of bacteremia includes prompt initiation of antibiotics at an appropriate dose based on a priori knowledge of guidelines, prompt microbial identification, and source control wherever possible. Empiric coverage may include piperacillin-tazobactam or cefepime plus metronidazole or a carbapenem (if concern for ESBL) with vancomycin or daptomycin. The recommended duration is 7 days for Gram-negatives and coagulase-negative staphylococci, and 14 days for MRSA and *Candida* species, and longer for complicated and resistant infections, immunocompromised patients, as well as endocarditis and osteomyelitis, as shown in table 3. For *S. aureus* bacteremia, an ID consultation should be considered. Stepdown to oral antibiotics is appropriate based on organism identified, severity of illness, and suspected source.

#### Discussion

Early and adequate treatment of bacteremia is essential. In general, higher doses of antibiotic are required early in the treatment course. Distinguishing community-acquired versus healthcare-acquired bacteremia is important to dictate antibiotic management. Previous antibiotic therapy, local antibiograms, and pharmacokinetic knowledge are important. The utilization of pharmacist expertise is critical for the provision of the best care in this circumstance. The specific antimicrobial treatment should be inspired by the primary source of infection in cases of secondary bacteremia.^71^ Early identification of the microbe, its sensitivities, and targeting of the antibiotic is important. There should be a high pretest probability for bacteremia prior to drawing blood cultures because the risk of contamination of blood culture specimens (false positive) remains significant. ‘Double coverage’ for Gram-negative bacteremia is no longer routinely recommended.^72 73^ An antifungal agent may be initiated depending on the clinical presentation and previous knowledge of *Candida* colonization. Where source control is obtained, the patient is clinically improved, and an appropriate oral antibiotic with favorable efficacy for the microbe is used, there is sufficient evidence to recommend stepdown to oral antibiotics to complete the total antibiotic course.^74 75^

There is now sufficient evidence to recommend 7 days of antibiotic therapy in cases of coagulase-negative staphylococci and Gram-negative bacteremia, 14 days for MRSA (unless a complicated infection is present as described in the section on CRBSI), and 14 days for *Candida*. Patients with candidemia require screening for retinitis if they are symptomatic for vision disturbance, are non-verbal, or have risk factors for ocular involvement (risk factors include long intravascular lines, parenteral nutrition, prolonged hospital stays, and recent abdominal surgery).^76^ These criteria based on recent data, reviews, and statements by the Royal College of Ophthalmologists and the American Academy of Ophthalmology have not yet been evaluated by the IDSA.^77 78^ Patients with ocular involvement should have an infectious disease consultation.

Follow-up blood cultures are not routinely needed for bacteremia and are discouraged except for S*. aureus* infections or in patients lacking clinical response.^79^ In difficult-to-treat infections, infectious disease consultation is advisable. Intravenous beta-lactam antibiotics are the best antibiotics for initial management of methicillin-susceptible *S. aureus*. Removal an infected device or a device suspected to be infected must be considered. Appropriate durations of therapy must be prescribed. Of note, an infectious disease consultation is of benefit for S*. aureus* bacteremia as it reduces morbidity and mortality, even in relatively minor infections.^80 81^

### Surgical site infections

#### Question

What are the treatment and antibiotic use recommendation for surgical site infection (SSI)?

#### Recommendation

The treatment of an SSI involves evacuation of infected material and one to 1–2 days of antibiotics if cellulitis is >5 cm or if significant systemic symptoms are present.

#### Discussion

The most important therapy for a patient with an SSI is prompt source control by removing the infected material. In superficial SSIs, removing sutures/staples may accomplish this. If the surrounding erythema is minor and the patient has no significant systemic symptoms, antibiotics are unnecessary. Otherwise, a short course of antibiotics (24–48 hours) may be appropriate, such as cefazolin for clean procedures and ceftriaxone plus metronidazole for intra-abdominal procedures.^82^ Persistence or recurrence of superficial signs/symptoms may indicate a deep or organ space SSI.

### Intra-abdominal infection

#### Question

In patients with IAI, what is the treatment and duration?

#### Recommendation

Initial antibiotic selection for IAI should be based on the source of infection, local antibiogram, and clinical severity. One reasonable empiric regimen is piperacillin-tazobactam for high-risk patients (plus vancomycin or linezolid in healthcare-associated IAI and ceftriaxone plus metronidazole for low-risk patients. Uncomplicated IAI (uIAI) can be managed with a single dose of preoperative antibiotic or a maximum of 24 hours postoperatively. Complicated IAI (cIAI) can be managed with 4 days of antibiotics once source control is achieved. When source control is not possible, we recommend 5–7 days of antibiotics. There is no demonstrated benefit in empiric antifungal therapy.

#### Discussion

uIAI include uncomplicated appendicitis or acute cholecystitis, traumatic bowel perforations managed within 12 hours, gastroduodenal perforations operated on within 24 hours, and resected ischemic bowel. cIAI are any IAI that extend beyond the site of origin or include the peritoneum. Treatment should involve prompt source control including emergent or urgent surgical exploration commensurate with the level of illness. Percutaneous options can be used if they achieve good source control. Any delay >24 hours is a predictor of failure and should be avoided. In systemically ill patients or patients with sepsis, initial blood cultures are indicated. Fluid or tissue from the source control procedure should be obtained to target antimicrobial selection.^83^

Antimicrobial therapy should be initiated as soon as an IAI is diagnosed or considered likely. The selection of antibiotics should be based on the local antibiogram and guided by a combination of culture results and the patient’s clinical status. Empiric antibiotics for severe community-acquired IAIs should include broad-spectrum Gram-negative coverage.^83^ Anaerobic coverage is also needed for which metronidazole is a recommended regimen, while for patients receiving piperacillin-tazobactam metronidazole is not necessary. Dual anerobic coverage is not recommended (except in specific infections including complicated *C. difficile* infections with vancomycin plus metronidazole and toxic shock syndrome for which treatment includes both vancomycin and clindamycin). In healthcare-associated infections, patients should be covered for MRSA such as with vancomycin or linezolid.^84^ High-risk patients should be given enterococcal coverage such as with vancomycin if they are not being treated with piperacillin-tazobactam.^84^

Patients with uIAI can be managed with either a single dose of perioperative antibiotic or a maximum of 24 hours of therapy.^83^ For cIAI, the most recent guidelines from the IDSA in 2010 and the Surgical Infection Society (SIS) in 2017 recommend shorter courses of antibiotics in patients who have adequate source control, 4–7 days and 4 days, respectively. The Study to Optimize Peritoneal Infection Therapy (STOP-IT trial) concluded that 4 days is sufficient.^85^ The SIS recommends a short 5–7 days course in patients without adequate source control with a reassessment of potential source control if the patient remains ill.^84^ We concur that there has not been evidence that describes a situation of IAI where courses >7 days are recommended, even in the presence of intraperitoneal sources with secondary bacteremia, and we agree that the emphasis is on thorough diagnostic evaluation and consideration for additional procedures when there is suspected failure of source control.

Empiric/Prophylactic preoperative antifungal therapy is not needed^86^ and routine post operative antifungal therapy in average risk patients is not recommended either.^87^ Only in high-risk patients, patients with prolonged perforation or preceding risk factors such as high-risk upper gastrointestinal perforations, recurrent bowel perforations, surgically treated pancreatitis, or prolonged antibiotic therapy can benefit from antifungal therapy.^84^ Additionally, the *Candida* score remains a useful tool, however, we recommend it be applied in the context of the data from the SIS. For instance, promptly treated bowel perforation in the absence of other risk factors should not be counted as a reason toward empiric fungal therapy based on recent evidence.^88 89^

### Ventriculitis

#### Question

In patients with ventriculitis, what is the most appropriate treatment?

#### Recommendations

A common antibiotic regimen for ventriculitis consists of vancomycin plus cefepime to cover hospital-acquired organisms. Removal of foreign body (ventricular shunt or external ventricular drain (EVD) may aid bacterial clearance. The duration of treatment is generally 10−14 days and can be longer for recurrent culture positivity or for *S. aureus*.

#### Discussion

Ventriculitis in hospitalized patients most commonly occurs in association with neurosurgical procedures, trauma resulting in dural tears and cerebrospinal fluid (CSF) leaks, or the insertion of a central nervous system (CNS) device such as a shunt or EVD. Diagnosis involves the biochemical profile and cultures of CSF and sometimes imaging such as CT or MRI to detect complications of ventriculitis including abscess. The organism may not always grow on culture media or may grow in a delayed fashion so the clinical context must be closely considered. Attention to CSF penetration must be given in antibiotic selection. In cases refractory to systemic antimicrobials, limited data support consideration of intraventricular administration.^90^ Specific recommendations about device-related infections, relative need for neuroimaging, and timeline of removal and re-implantation are beyond the scope of this article.

### Necrotizing soft tissue infection

#### Question

In patients with necrotizing soft tissue infection (NSTI), what is the preferred antibiotic therapy and duration?

#### Recommendation

Empiric antibiotic therapy for NSTIs should be broad-spectrum and reflect local resistance patterns. First-line treatments include linezolid or (vancomycin plus clindamycin in combination with piperacillin-tazobactam.

We recommend 2–4 days of antibiotics after final debridement if the following conditions are met: (1) favorable wound appearance, (2) subjective clinical improvement, (3) no fever for 48 hours after last debridement, (4) relative improvement of laboratory values (white blood cell, lactate, etc) and (5) the initial blood cultures are negative. We recommend 5–7 days of therapy in patients who meet sepsis (sepsis-3) or have septic shock that does not improve after initial resuscitation or who did not have blood cultures drawn at presentation. Patients with marine or fresh water exposure require special antibiotic considerations, as described in the discussion.

#### Discussion

Initial therapy should include coverage for Gram-positive and Gram-negative (aerobic and anaerobic) organisms including MRSA, as well as group A and group B streptococci. Antibiotics chosen should have good tissue penetrance, especially since many patients with NSTI have severe diabetes.

Piperacillin-tazobactam has broad Gram-negative aerobic coverage which is a gap in coverage with linezolid and microbes causing NSTI continue to have good sensitivity.^91^

Linezolid has recently been shown to be associated with better clinical and microbiological cure rates than vancomycin.^92^ Because linezolid has antitoxin activity, it obviates the need for clindamycin.^93^ Furthermore, linezolid is more likely to cover group B streptococci which are common in NSTI.^94^ Clindamycin has a long history of clinical data and there is not enough data to discern whether the rising resistance in group A *Streptococcus* is clinically significant. Clinical superiority for linezolid is suggested in NSTI caused by MRSA,^93^ although the most recent evidence does not show overall clinical superiority.^95^ Linezolid is associated with less acute kidney injury than the alternative treatment with vancomycin and a shorter hospital length of stay but a higher risk of thrombocytopenia.^96^ Vancomycin plus clindamycin remain a different but equal choice as linezolid as the debate continues.^97^

Rare but important exposures that can lead to fatal infections are the following: (1) marine exposure—the antibiotic regimen must then cover vibrio species, namely (a quinolone or a tetracycline) plus a third-generation cephalosporin.^98^ Exposure to fresh water, soil, wood—the antibiotic regimen must then cover *Aeromonas*, namely a tetracycline with either ciprofloxacin or ceftriaxone, based on known effectiveness of these agents.^82 99^ We do not advise carbapenems because there are rising reports of resistance.^100–102^

Regarding the duration of antibiotics, we base our current antibiotic recommendation on recent literature and recommend a 2-day to 4-day course provided the conditions above are met. The presence of initial negative blood cultures would obviate the need to treat a bacteremia; furthermore, given that patients with positive blood culture were excluded from the index study, one cannot conclude that patients without initial blood culture data are safe to be included in the short-course treatment strategy.^103 104^

## Conclusion

Evaluating fever and determining the likelihood of an underlying infection can be challenging. It is important for the surgical intensivist to remain vigilant to identify sepsis and septic shock and also to exercise clinical judgment and forethought when ordering antibiotics. In the absence of sepsis (as defined by sepsis-3 guidelines) and septic shock, the data support selective utilization of cultures and antibiotic use. In most infections, the evidence is accumulating in favor of the safety and efficacy of shorter courses of treatment. Table 3 provides a summary of the recommendations for antibiotic durations for common ICU infections. We present here a consensus summary from the AAST Critical Care Committee for our approach to fever in the ICU and for the treatment of common surgical intensive care infections, namely VAP, UTI, CRBSI, bacteremia, intra-abdominal abscess, SSI, ventriculitis, and necrotizing skin and soft tissue infections.
